# Therapeutic potential of maggot-derived bioactive peptides in modulating inflammatory responses in mice model of inflammatory bowel disease

**DOI:** 10.3389/fcell.2026.1976594

**Published:** 2026-09-25

**Authors:** Lina Zhou, Yan Chen, Mingfeng Liu, Mei Wu, Haojie Deng, Zhiwei Miao, Xiaoyu Wang, Chunyu Zhang, Ke Hua, Chanchan Guan, Jingjing Cao

**Affiliations:** 1 Department of Surgery, Zhangjiagang TCM Hospital Affiliated to Nanjing University of Chinese Medicine, Zhangjiagang, China; 2 The Affiliated Taizhou People’s Hospital of Nanjing Medical University, Taizhou, China; 3 The Affiliated Zhangye People’s Hospital of Hexi University, Zhangye, China; 4 Center of Reproductive Medicine, Jiaxing Maternity and Child Healthcare Hospital, College of Medicine, Jiaxing University, Jiaxing, China; 5 Department of Gastroenterology, Jiangsu Province TCM Hospital Affiliated to Nanjing University of Chinese Medicine, Nanjing, China

**Keywords:** BPM, inflammation, inflammatory bowel disease, mucosal barrier, TNF-α

## Abstract

**Introduction:**

Inflammatory bowel disease (IBD) is characterized by chronic inflammation and disruption of intestinal integrity. This study investigates the therapeutic effects of Maggot-Derived Bioactive Peptides (BPM) in alleviating IBD symptoms and restoring intestinal function.

**Methods:**

Using an IBD model, we assessed the impact of BPM on inflammatory responses, intestinal morphology, goblet cell density, and tight junction protein expression. Serum levels of inflammatory and oxidative stress markers were also measured to evaluate systemic effects.

**Results:**

BPM significantly alleviated inflammatory responses in IBD, as evidenced by decreased levels of pro-inflammatory cytokines, including TNF-α. Treatment with BPM enhanced intestinal morphology and increased goblet cell density, promoting mucosal barrier function. Furthermore, BPM regulated p-p65 and TNF-α pathways, effectively mitigating inflammation. Serum analysis revealed that BPM modulated the expression of inflammatory and oxidative stress markers. Importantly, BPM preserved intestinal wall integrity by upregulating tight junction proteins ZO-1, occludin, and claudin-1.

**Discussion:**

Our findings suggest that BPM is a promising therapeutic agent for IBD, exerting anti-inflammatory effects, enhancing intestinal morphology, and preserving epithelial integrity through multiple mechanisms. Further investigations are warranted to explore BPM’s potential as a standard treatment option for IBD.

## Introduction

Inflammatory bowel disease (IBD) is a chronic and relapsing inflammatory condition of the gastrointestinal tract, comprising primarily Crohn’s disease and ulcerative colitis (UC), and its prevalence has been increasing globally in recent decades (Gajendran et al., 2018; Kuenzig et al., 2022; Kuenzig et al., 2022). Despite advances in therapeutic approaches, the etiology of IBD remains elusive, and current treatments often present limitations, including adverse side effects and reduced efficacy over time (Lefevre and Vande Casteele, 2020). Inflammation in IBD is marked by an excessive immune response that involves a complex interplay of proinflammatory cytokines, oxidative stress, and mucosal immune dysregulation, ultimately leading to tissue injury and compromised gut barrier integrity (Saez et al., 2023; Zhao et al., 2024). Reactive oxygen species (ROS) and oxidative stress are central mediators in this pathogenesis, contributing to epithelial apoptosis and persistent inflammation, both of which exacerbate intestinal mucosal damage (Redza-Dutordoir and Averill-Bates, 2016). The enzymes superoxide dismutase (SOD) and catalase (CAT) are essential antioxidant defenses within the gut, and their activity is typically diminished in IBD, reflecting oxidative imbalance (Ashique et al., 2023). Therefore, therapeutic strategies that target oxidative stress and modulate inflammatory pathways could offer significant promise for the effective management of IBD (Kim and Chang, 2014; Khan et al., 2019).

Maggot-derived bioactive peptides (BPM) represent a unique class of naturally occurring compounds with promising therapeutic properties. Farmed maggots, a natural edible, is fit for human consumption such as sauces in China. On the other hand, maggot therapy has been employed as an effective intervention for wound healing, due to its demonstrated antimicrobial and anti-inflammatory effects, especially in treating skin infections and necrotic tissue (Moya-López et al., 2020; Stadler, 2020). Larvae of *Lucilia sericata*, commonly known as maggots, are rich in proteins, amino acids, and essential fatty acids, which contribute to their potent bioactivity (Tantawi et al., 2010; Morozov and Sherman, 2019). Studies have shown that bioactive peptides from various insects exert antioxidative, anti-inflammatory, and immune-modulatory effects, which are relevant to IBD pathology (Wang et al., 2021). For instance, bioactive compounds from cricket and other insects have shown potential in modulating metabolic and inflammatory pathways, suggesting that insect-derived peptides may have utility in treating inflammation-driven disorders (Dutta et al., 2019).

Given the promising biological properties of maggot-derived peptides, this study aims to investigate the potential therapeutic effects of BPM in a murine model of IBD. Specifically, we explore whether BPM can modulate oxidative stress and inflammatory signaling through key pathways, focusing on markers such as TNF-α, NF-κB, and tight junction proteins like ZO-1 and occludin, which play critical roles in maintaining intestinal barrier integrity and regulating immune responses in intestinal inflammation (Xiong et al., 2022; Yang Q et al., 2024). By elucidating these mechanisms, our study highlights the potential of BPM as an innovative, bioactive therapeutic approach for reducing inflammation and oxidative damage in IBD, with implications for improving treatment outcomes in patients with this challenging disease.

## Materials and methods

### Preparation of BPM

BPM, with the amino acid sequence Gly-Tyr-Gly-Gly-Tyr-Gly-Gly-Phe-Gly-Arg, was synthesized as part of this study. The sequence and amino acid composition were provided by the State Key Laboratory of Analytical Chemistry for Life Science and the Jiangsu Key Laboratory of Molecular Medicine at Nanjing University. Following synthesis, the peptide underwent purification to ensure high quality. The purity of the final product was assessed using high-performance liquid chromatography (HPLC), which confirmed that it exceeded 99%. This high level of purity is critical for ensuring the reliability of subsequent experiments and validating the therapeutic effects of BPM in the context of inflammatory bowel disease.

### IBD model establishment and animal ethics

A total of 40 male C57BL/6 mice, aged 8–10 weeks, were obtained from GemPharmatech Co., Ltd (China). The mice were housed in a specific pathogen-free (SPF) environment at a controlled temperature of 25 °C with 50% relative humidity and a 12-h light/dark cycle. They had free access to food and water throughout the experimental period, except that Dextran sodium sulfate (DSS, 36–50kDa, MP Biomedicals, USA)-treated mice received DSS-containing drinking water during the induction phase. To induce inflammatory bowel disease (IBD) models, mice received 3% (w/v) DSS in drinking water for 7 days, followed by regular drinking water until day 10. Then mice were randomized into four groups (n = 10 per group): the normal group, the IBD group, the IBD group treated with BPM (IBD + BPM), and the IBD group treated with Mesalazine (IBD + Mesalazine) (Mizoguchi, 2012). BPM was freshly dissolved in deionized water and administered by oral gavage once daily at a dose of 20 mg/kg in a volume of approximately 100 µL per mouse. No protective carrier, encapsulation system, or enteric formulation was used. Mice in the IBD + Mesalazine group received Mesalazine (100 mg/kg/day) by oral gavage once daily during the treatment period.

All animal experiments were conducted in compliance with the ARRIVE guidelines and adhered to the United Kingdom. Animals (Scientific Procedures) Act, 1986, the European Communities Council Directive 2010/63/EU, and the policies set forth by the National Institutes of Health–Office of Laboratory Animal Welfare. The Ethics Committee of Nanjing Normal University approved this study (IACUC-20230249). Furthermore, all procedures followed the guidelines established by the China Council on Animal Care and Use. Inhumane treatment was strictly avoided, with such measures applied only when absolutely necessary.

### Histological examination of colonic tissue

Following fixation in 4% paraformaldehyde, colonic tissue samples were embedded in paraffin and sectioned at a thickness of 4 µm. The sections were then deparaffinized and stained with hematoxylin and eosin (H&E) to evaluate general tissue morphology and with Periodic Acid–Schiff (PAS) stain to specifically visualize goblet cells. Histological scoring of tissue damage in H&E-stained sections was conducted to assess pathological changes, including epithelial disruption, crypt damage, and inflammatory cell infiltration. Finally, the structural integrity and goblet cell density in the colonic tissue were analyzed based on the stained slides. Histological evaluation was performed independently by two observers who were blinded to the treatment groups. The scores for epithelial disruption, crypt damage, and inflammatory cell infiltration were compared between observers, and any discrepancies were resolved by joint review to reach a consensus score for final statistical analysis.

### Immunofluorescence staining

Tissue sections (4–6 µm thick) were prepared from paraffin-embedded, 10% formalin-fixed samples. Following deparaffinization with xylene and rehydration through graded ethanol, nonspecific binding was blocked by incubating sections in 1% bovine serum albumin (BSA) in PBS/Tween. Primary antibodies against occludin (91,131, CST), ZO-1 (ab221547, Abcam), p-p65 (3033, CST) and TNF-α (11,948, CST) were applied (diluted 1:500), and sections were incubated overnight at 4 °C. The following day, sections were exposed to secondary antibodies (1:1000) for 30 min at room temperature in darkness. Nuclei were counterstained with DAPI, and imaging was conducted using an Olympus FV3000 laser-scanning confocal microscope. For immunofluorescence quantification, images for each marker were acquired under identical microscope settings, including laser intensity, gain, exposure, and magnification. For each mouse, five randomly selected non-overlapping fields from the colonic mucosal area were analyzed using ImageJ software. The fluorescence signal of the target protein was measured in the green channel after background subtraction, and the mean fluorescence intensity (MFI) was calculated as integrated density divided by the selected tissue area. Field-level values were averaged to obtain one value for each mouse, and the individual mouse was used as the experimental unit for statistical analysis. Relative MFI values were normalized to the mean value of the Normal group.

### Evaluation of oxidative stress status and inflammation factors

This study assessed the oxidative stress status of the colons by measuring the levels of superoxide dismutase (SOD), glutathione peroxidase (GSH-Px), and myeloperoxidase (MPO) in the tissue. The samples were then weighed, homogenized, and diluted to 10% (v/v) in an ice bath. After centrifugation at 600 *g* for 15 min at 4 °C, the supernatants were collected for the measurement of SOD, GSH-Px, and MPO levels using a commercial kit (Nanjing Jiancheng Bioengineering Institute, Nanjing, China) according to the manufacturer’s instructions.

Additionally, inflammation factors including IL-1β, IL-6, TNF-α, IFN-γ, and IL-10 were quantified using enzyme-linked immunosorbent assay (ELISA) methods. Samples were processed according to the instructions provided in the ELISA kits to ensure accuracy and reproducibility of the results.

### Western blot analysis

Total protein extracted from colon samples was separated on 10% SDS-PAGE and transferred to PVDF membranes (Millipore, Boston, MA, USA). Membranes were then blocked in 5% bovine serum albumin in TBST and incubated overnight at 4 °C with primary antibodies against occludin, ZO-1, claudin-1 and GAPDH (1:2000; Abcam, USA). The following day, membranes were incubated with goat anti-rabbit secondary antibody (1:40,000) and washed three times before bands were visualized using a chemiluminescent substrate (Vazyme Biotech, Nanjing, China). Protein band intensities were quantified with ImageJ software, with all target proteins normalized to GAPDH.

### Statistical analysis

Statistical analyses were performed using GraphPad Prism 9.0 (GraphPad Software, San Diego, CA, USA). Data are presented as the mean ± standard deviation (SD), and the individual mouse was treated as the experimental unit. Longitudinal body-weight data were analyzed using two-way repeated-measures ANOVA, with group and time as factors, followed by multiplicity-adjusted *post hoc* comparisons. Single-time-point continuous outcomes involving multiple groups were analyzed using one-way ANOVA followed by Dunnett’s multiple-comparisons test, with the DSS group as the reference. Ordinal outcomes, including the terminal Disease Activity Index (DAI) and histological scores, were analyzed using the Kruskal–Wallis test followed by Dunn’s multiple-comparisons test. Measurements obtained from multiple microscopic fields or technical replicates were averaged within each mouse before group-level statistical analysis. P < 0.05 was considered statistically significant. All data represent findings from three or more independent experiments.

## Results

### BPM alleviates inflammatory responses in inflammatory bowel disease (IBD)

The therapeutic effects of BPM on disease progression in a mouse model of inflammatory bowel disease (IBD), with comparisons to a mesalazine-treated positive control group and untreated controls (Figure 1). The IBD induction procedure in mice, including the timeline and specific steps for establishing the experimental model (Figure 1A).

**FIGURE 1:**
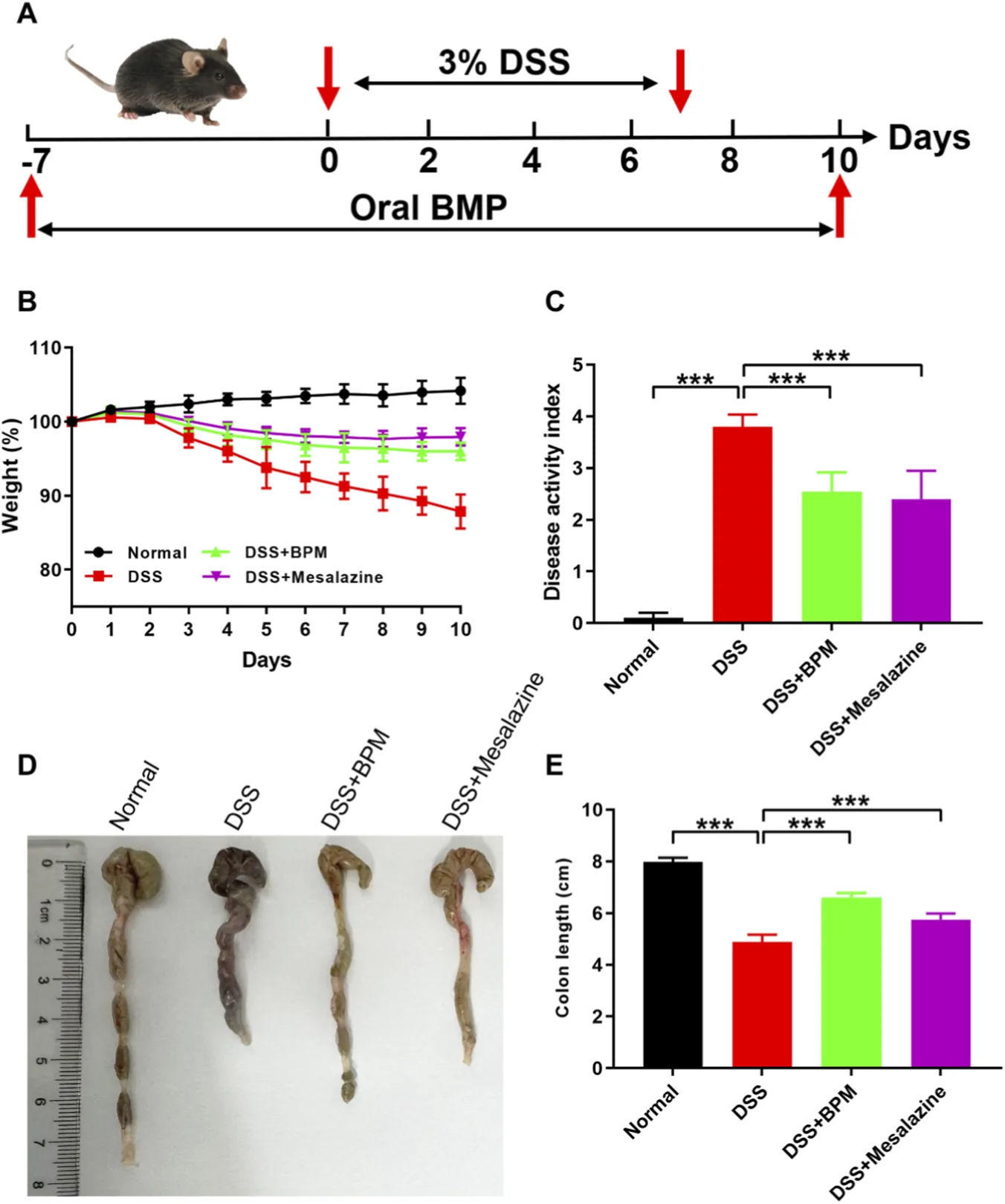
BPM ameliorated the clinical symptoms in IBD mice. **(A)** Experimental design. **(B)** Body weight change. **(C)** DAI scores. **(D)** Macroscopic features of the colon. **(E)** Results of colonic length quantification. Data presented are means ± SD. *P < 0.05, **P < 0.01, ***P < 0.001, ns = no significance.

The body weight trajectories across the four groups (Normal control, IBD model, BPM treatment, and mesalazine treatment). Mice in the IBD model group experienced a marked decline in body weight over the observation period, reflecting the physiological burden of induced colitis (Figure 1B). In contrast, both the BPM and mesalazine treatment groups displayed a significantly mitigated weight loss relative to the untreated IBD model group, suggesting that BPM treatment contributes to maintaining body weight stability under inflammatory conditions (p < 0.05) (Figure 1B).

The Disease Activity Index (DAI) scores (Liu et al., 2020), a composite metric for assessing disease severity based on weight loss, stool consistency, and rectal bleeding (Figure 1C). DAI scores in the IBD model group were significantly elevated compared to the normal control group, underscoring the effective induction of IBD symptoms in this model (p < 0.01). Notably, both BPM-treated and mesalazine-treated groups demonstrated a significant reduction in DAI scores relative to the untreated IBD model (p < 0.05), indicating that BPM effectively alleviates key clinical manifestations of colitis. These results collectively suggest that BPM exhibits anti-inflammatory and protective effects, similar to mesalazine, in mitigating IBD-associated weight loss and reducing DAI scores, highlighting its potential as a therapeutic agent for IBD.

The BPM treatment significantly ameliorated colonic shortening. Compared to the DSS group, the colons of BPM-treated mice exhibited a marked recovery in length, indicating a protective effect of BPM against DSS-induced colonic damage (Figures 1D,E).

### BPM significantly enhances intestinal morphology and increases goblet cell density in the treatment of inflammatory bowel disease

In Figure 2A, histological examination of colonic tissue sections from the inflammatory bowel disease (IBD) mouse model was performed using Hematoxylin and Eosin (H&E) staining and Periodic Acid–Schiff (PAS) staining. The H&E-stained sections highlight that BPM treatment significantly restores colonic morphology. In untreated IBD model mice, extensive mucosal damage was observed, characterized by epithelial disruption, crypt loss, and inflammatory cell infiltration. However, BPM-treated mice show marked improvements, including preservation of epithelial architecture, reduced inflammatory infiltration, and better-defined crypt structures. These observations suggest that BPM treatment mitigates structural damage in the colon caused by IBD, contributing to the restoration of tissue integrity. Quantitatively evaluates these histopathological changes through a scoring system based on H&E-stained sections (Figure 2B). Mice in the BPM treatment group exhibit significantly lower histopathological scores compared to untreated IBD model mice, indicating a reduction in overall pathological damage. This decrease in damage scores highlights the protective role of BPM in preserving colonic tissue architecture in the IBD model.

**FIGURE 2:**
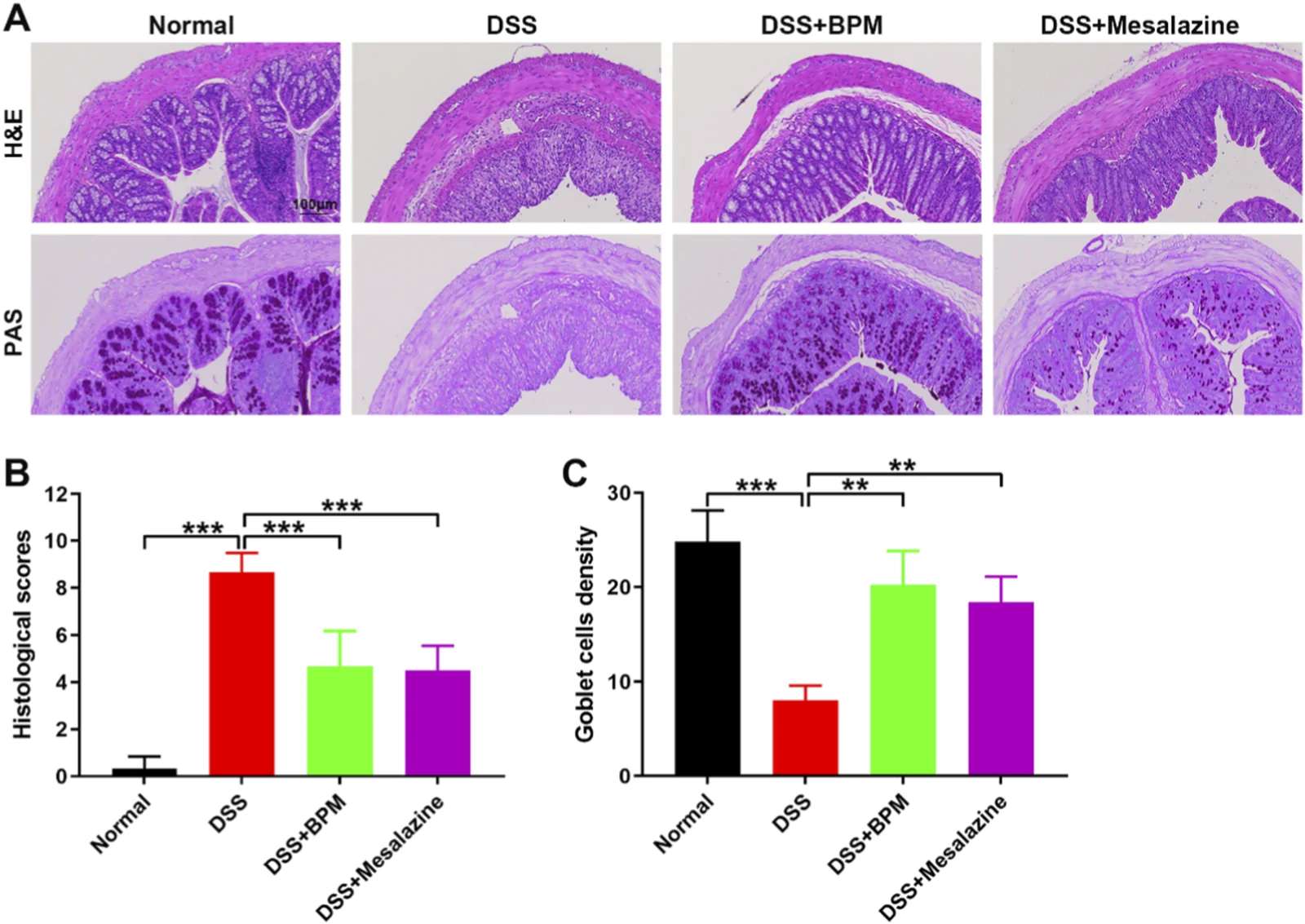
Effects of BPM on histopathological changes in IBD mice. **(A)** Representative images of H&E and PAS staining in the colon. **(B)** Histological scores. **(C)** Goblet cell density. Data presented are means ± SD. *P < 0.05, **P < 0.01, ***P < 0.001, ns = no significance.

Further analysis of PAS-stained sections in Figure 2A and the quantification of goblet cell density reveal that BPM treatment enhances goblet cell populations, which are critical for mucus production and intestinal barrier function (Figure 2C). Untreated IBD model mice show a noticeable depletion of goblet cells, indicating compromised mucosal barrier protection. In contrast, BPM-treated mice display a significant increase in goblet cell density, as quantified in Figure 2C. This increase suggests that BPM promotes mucosal repair and strengthens barrier integrity by restoring goblet cell numbers, potentially enhancing mucus layer formation essential for gut health.

In summary, these findings that BPM treatment not only ameliorates morphological damage in the colonic tissue of IBD model mice but also enhances goblet cell density, potentially reinforcing mucosal defenses and reducing disease severity in IBD.

### BPM mediates IBD treatment by regulating p-p65 and TNF-α pathways and improving intestinal wall integrity

Fluorescence staining was used to assess the expression and localization of proteins critical for intestinal barrier integrity and inflammatory signaling in the IBD model following BPM treatment. The expression of occludin, a tight junction protein essential for intestinal barrier function. Fluorescence intensity in BPM-treated mice shows a marked increase in occludin expression along the intestinal wall compared to untreated IBD model mice, indicating enhanced barrier integrity (Figure 3A). Similarly, The expression of ZO-1, another key tight junction protein. BPM treatment results in a significantly intensified ZO-1 fluorescence signal, reflecting the reestablishment of tight junctions and improved mucosal barrier function, which is essential in protecting against inflammation-induced damage (Figure 3B).

**FIGURE 3:**
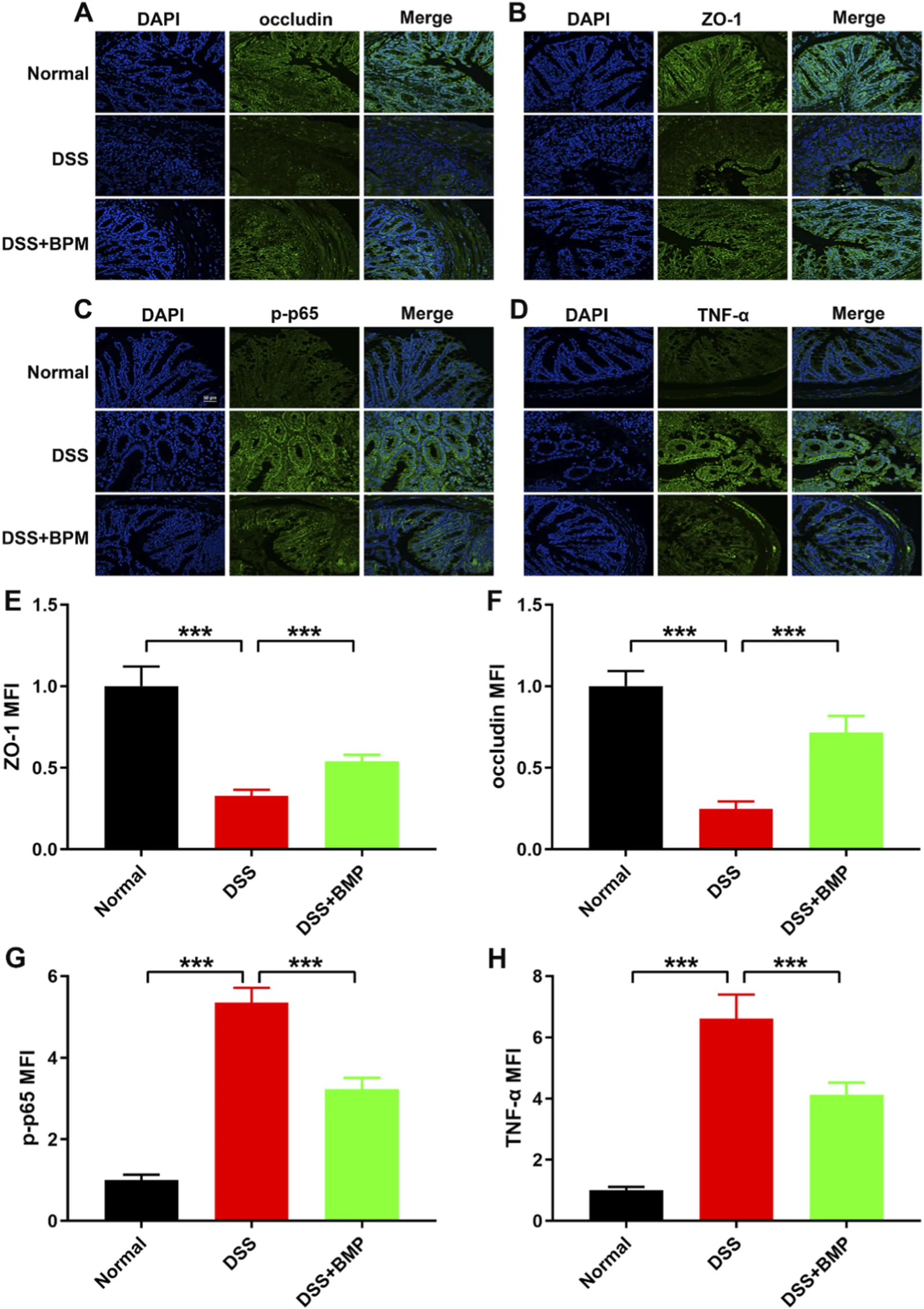
Effects of BPM on TJ protein and inflammatory index in IBD mice. **(A,B)** The expression of occludin and ZO-1 in colonic tissues was assessed by immunostaining. **(C,D)** The expression of p-p65 and TNF-α in colonic tissues was assessed by immunostaining. Inverted fluorescence microscopy was employed to capture the entire representative micrographs (scale bar = 100 μm) **(E–H)** Quantitative analysis of the mean fluorescence intensity (MFI) of ZO-1, occludin, p-p65, and TNF-α. Quantification was performed using ImageJ, and field-level measurements were averaged within each mouse before statistical analysis. Data presented are means ± SD. *P < 0.05, **P < 0.01, ***P < 0.001, ns = no significance.

The phosphorylation levels of p65 (p-p65), a key protein in the NF-κB signaling pathway, which plays a pivotal role in regulating inflammation. In untreated IBD model mice, p-p65 fluorescence intensity is significantly elevated, indicating increased NF-κB pathway activation and heightened inflammatory response (Figure 3C). However, BPM treatment notably reduces p-p65 levels, as evidenced by decreased fluorescence intensity, suggesting that BPM downregulates NF-κB activation and thereby mitigates inflammation within the intestinal tissue (Figure 3C).

Additionally, we focused on the expression of TNF-α, a pro-inflammatory cytokine heavily involved in the pathogenesis of IBD. In the untreated IBD model, TNF-α fluorescence intensity is elevated, reflecting an inflammatory response that likely contributes to tissue damage. Following BPM treatment, TNF-α expression is significantly reduced, as indicated by lower fluorescence intensity in the BPM-treated samples (Figure 3D). This reduction implies that BPM suppresses TNF-α production, potentially lowering inflammation levels and contributing to improved tissue conditions. Quantitative analysis of immunofluorescence intensity further confirmed the staining observations. Compared with the IBD group, BPM treatment significantly increased the fluorescence intensities of occludin and ZO-1, while significantly reducing the fluorescence intensities of p-p65 and TNF-α.

In summary, these findings suggest that BPM treatment improves intestinal wall integrity by upregulating tight junction proteins occludin and ZO-1, while simultaneously reducing inflammatory signaling through the downregulation of p-p65 and TNF-α pathways. These combined effects highlight BPM’s potential therapeutic mechanism in IBD, targeting both barrier integrity and inflammation regulation.

### BPM modulated the expression of inflammatory and oxidative stress markers

We detected the serum levels of key inflammatory and oxidative stress markers in the IBD model, comparing BPM-treated mice to untreated controls (Figure 4). The levels of inflammatory cytokines in colonic tissues were further evaluated. Compared with the Normal group, DSS treatment significantly increased the pro-inflammatory cytokines IL-1β, IL-6, TNF-α, and IFN-γ, while reducing the anti-inflammatory cytokine IL-10. BPM administration markedly decreased IL-1β, IL-6, TNF-α, and IFN-γ levels and restored IL-10 expression compared with the IBD group. Similar protective effects were observed in the Mesalazine-treated group. These results indicate that BPM alleviates DSS-induced intestinal inflammation by suppressing pro-inflammatory cytokine production and improving the anti-inflammatory cytokine response.

**FIGURE 4:**
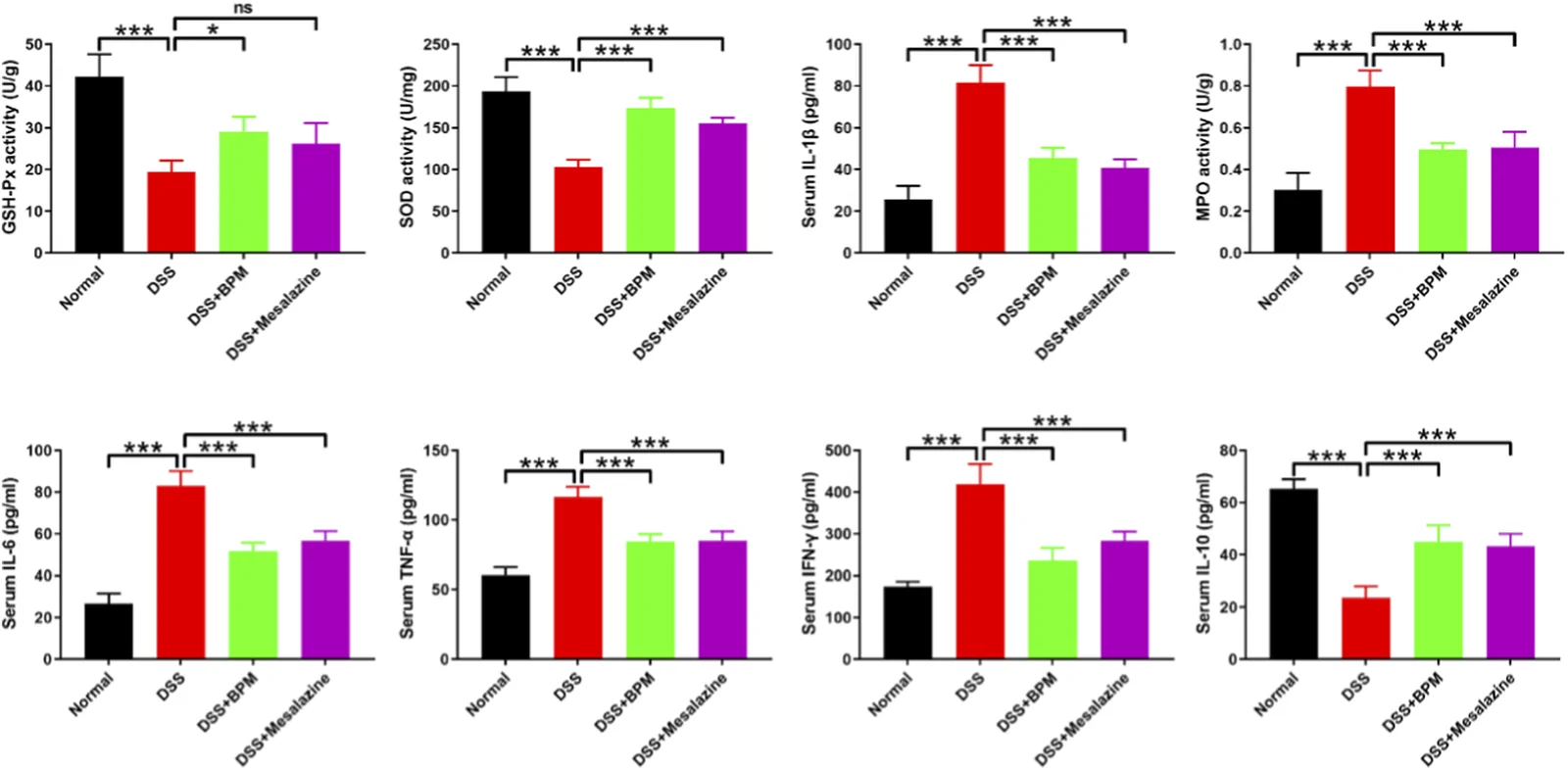
BPM modulated the expression of inflammatory and oxidative stress markers. BPM attenuated oxidative stress and inflammatory responses in DSS-induced colitis mice. The levels of oxidative stress-related indicators and inflammatory cytokines, including GSH-Px, SOD, MPO, IL-1β, IL-6, TNF-α, IFN-γ, and IL-10, were measured from the Normal, IBD, IBD + BPM, and IBD + Mesalazine groups. Data presented are means ± SD. *P < 0.05, **P < 0.01, ***P < 0.001, ns = no significance.

Conversely, levels of antioxidant enzymes such as superoxide dismutase (SOD) and glutathione peroxidase (GSH-Px) are reduced in untreated IBD model mice, likely due to increased oxidative burden (Tian et al., 2021). BPM treatment significantly restores the serum levels of these enzymes, suggesting an enhancement of the endogenous antioxidant defense system.

In summary, these ELISA results indicate that BPM modulates the expression of key inflammatory and oxidative stress markers in serum, reducing pro-inflammatory cytokines and oxidative damage markers while enhancing antioxidant enzyme levels. This profile suggests a dual anti-inflammatory and antioxidant mechanism by which BPM may exert therapeutic effects in IBD.

### BPM protected intestinal wall integrity in IBD by regulating ZO-1, occludin, and claudin-1 *in vivo*

In Figure 5A, Western blot analysis was used to assess the protein expression levels of ZO-1, occludin, and claudin-1, which are critical components of tight junctions essential for maintaining intestinal barrier integrity. In the untreated IBD model, these proteins are markedly downregulated, indicating disrupted tight junctions and impaired barrier function associated with intestinal inflammation. However, BPM treatment results in a notable upregulation of ZO-1, occludin, and claudin-1 levels, suggesting that BPM helps restore tight junction protein expression and potentially strengthens the integrity of the intestinal wall in the IBD model. The relative quantification of these tight junction proteins, further highlighting the significant differences in expression between BPM-treated and untreated IBD mice (Figure 5B). Quantitative analysis shows a statistically significant increase in the levels of ZO-1, occludin, and claudin-1 in the BPM-treated group compared to the untreated IBD group (p < 0.05). This result supports the notion that BPM treatment enhances intestinal barrier function by promoting the expression of key tight junction components, which are otherwise compromised in IBD.

**FIGURE 5:**
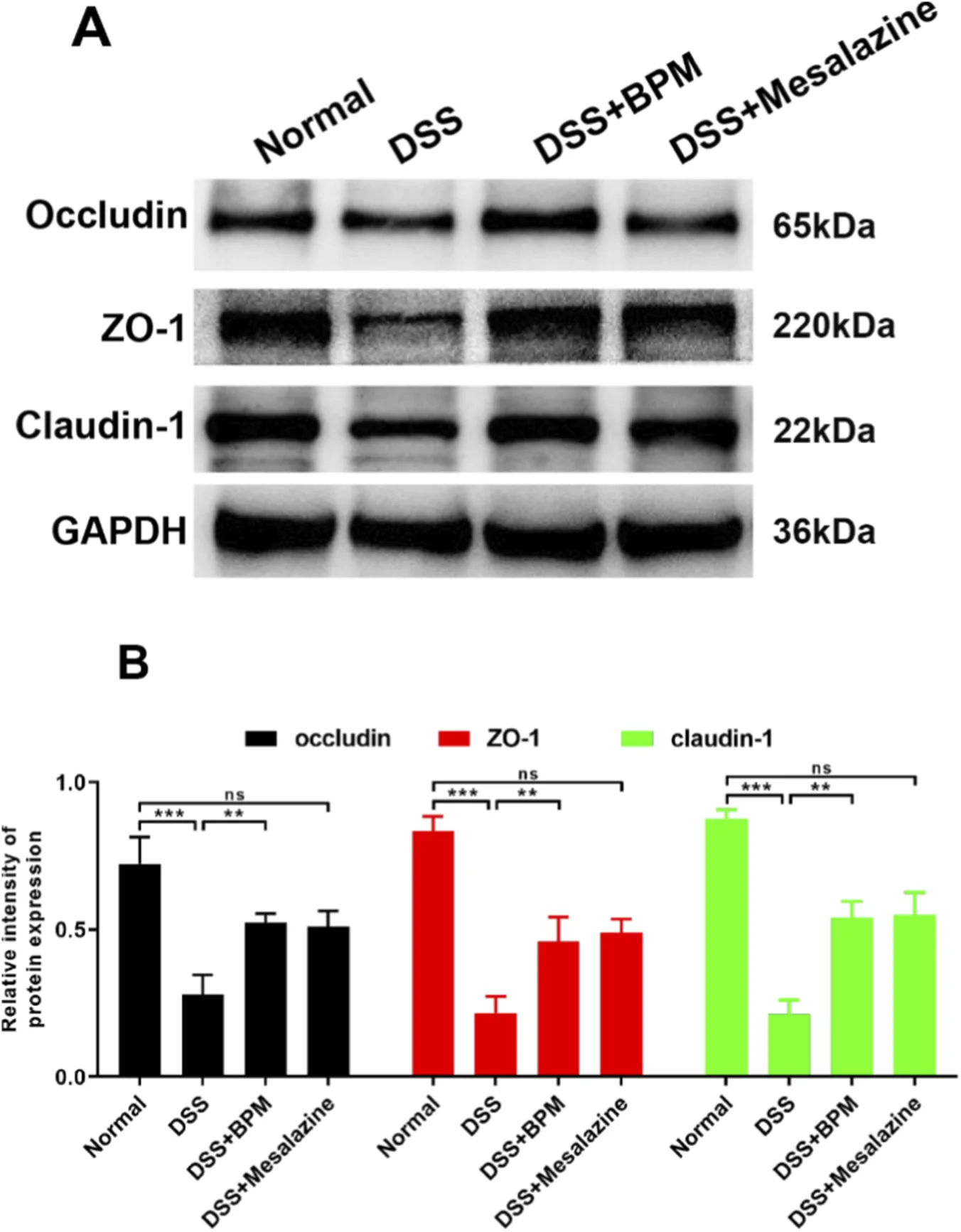
BPM protected intestinal wall integrity in IBD by regulating ZO-1, occludin, and claudin-1 *in vivo*. **(A,B)** The colonic tissue levels of ZO-1, occludin, and claudin-1 were detected by Western blotting. The relative intensities of these proteins were normalized to GAPDH. Data presented are means ± SD. *P < 0.05, **P < 0.01, ***P < 0.001, ns = no significance.

In summary, these findings suggest that BPM exerts protective effects on the intestinal wall by upregulating essential tight junction proteins, including ZO-1, occludin, and claudin-1, *in vivo*. This upregulation is indicative of BPM’s potential therapeutic role in reinforcing barrier integrity and mitigating damage in inflammatory bowel disease.

## Discussion

The present study investigates the therapeutic effects of BPM in inflammatory bowel disease (IBD), highlighting its multifaceted mechanisms of action. Our findings demonstrate that BPM alleviates inflammatory responses, significantly enhances intestinal morphology, and increases goblet cell density, ultimately contributing to the restoration of intestinal integrity (Dong et al., 2022).

Firstly, the ability of BPM to mitigate inflammatory responses in IBD is noteworthy. Chronic inflammation is a hallmark of IBD, often exacerbating tissue damage and impairing healing processes (Yang W et al., 2024). Our results indicate that BPM exerts a robust anti-inflammatory effect, likely through the modulation of critical signaling pathways. Specifically, BPM was found to regulate p-p65 and TNF-α pathways, which are pivotal in the inflammatory cascade (Yu et al., 2022). By downregulating these pro-inflammatory markers, BPM effectively reduces the inflammatory burden within the intestinal environment, which is essential for the management of IBD (Shen et al., 2022).

Secondly, the enhancement of intestinal morphology and the increase in goblet cell density observed with BPM treatment signify its protective role in maintaining intestinal homeostasis (Parikh et al., 2019; He et al., 2023). Goblet cells are crucial for mucus production, providing a protective barrier against luminal pathogens and maintaining epithelial integrity (He et al., 2023). The restoration of goblet cell density is particularly significant, as it correlates with improved mucosal barrier function, reducing the risk of further inflammation and damage (Benoit et al., 2015).

Furthermore, BPM’s modulation of inflammatory and oxidative stress markers in serum underscores its systemic effects on IBD (Sahoo et al., 2023). Elevated levels of oxidative stress and inflammation are commonly associated with IBD pathogenesis, contributing to epithelial damage and disease progression. By effectively reducing these markers, BPM not only aids in local intestinal repair but also offers systemic benefits that may enhance overall patient outcomes.

In addition, our study elucidates the role of BPM in preserving intestinal wall integrity through the regulation of tight junction proteins such as ZO-1, occludin, and claudin-1. These proteins are critical for maintaining the structural integrity of the intestinal epithelium. Disruption of tight junctions is often observed in IBD, leading to increased intestinal permeability and subsequent inflammation (Lee, 2015). BPM’s ability to upregulate these junction proteins suggests a mechanism by which it reinforces the epithelial barrier, mitigating the inflammatory processes that characterize IBD (Laurindo et al., 2023).

An important limitation of the present study is that the gastrointestinal stability, bioavailability, and luminal interactions of orally administered BPM were not directly evaluated. BPM was administered as an aqueous solution without a protective carrier or enteric formulation and may therefore have been exposed to gastric acidity and gastrointestinal proteases. Consequently, although oral BPM produced significant protective effects in the DSS-induced colitis model, the current data do not establish whether the intact peptide reached the colon or whether peptide fragments generated during gastrointestinal digestion contributed to the observed effects. In addition, because IBD is closely associated with gut microbial dysbiosis and orally administered bioactive peptides may interact with the intestinal microbiota, microbiota-dependent mechanisms may also participate in the protective effects of BPM. However, fecal microbiota sequencing and metabolomic analyses were not performed in this study; therefore, we cannot determine whether BPM altered gut microbial composition or microbial metabolites such as short-chain fatty acids, bile acids, or tryptophan-derived compounds. Future studies should assess the stability of BPM in simulated gastric and intestinal fluids, quantify intact BPM and its major degradation products using analytical methods such as liquid chromatography-tandem mass spectrometry, characterize its pharmacokinetics and tissue distribution, and investigate gut microbiota-related mechanisms using 16S rRNA gene sequencing, shotgun metagenomics, targeted metabolomics, microbiota-depletion models, or fecal microbiota transplantation. Protective delivery strategies, including enteric coating or encapsulation, should also be explored to improve gastrointestinal stability and colonic delivery.

In conclusion, our findings collectively demonstrate that BPM serves as a promising therapeutic agent for IBD, functioning through diverse mechanisms that encompass anti-inflammatory effects, enhancement of intestinal morphology, and preservation of epithelial integrity. Future studies should further elucidate the specific molecular pathways involved and explore the potential for BPM as a standard treatment option in clinical settings.

## Data Availability

The original contributions presented in the study are included in the article/supplementary material, further inquiries can be directed to the corresponding author/s.
